# Management of Native Joint Septic Arthritis, Serial Aspiration vs. Arthroscopic Washout During the COVID-19 Pandemic

**DOI:** 10.7759/cureus.11391

**Published:** 2020-11-09

**Authors:** Danielle Piper, Gemma Smith, James E Archer, Hugo Woffenden, Deepa Bose

**Affiliations:** 1 Trauma and Orthopaedics, Queen Elizabeth Hospital, Birmingham, GBR; 2 Trauma and Orthopaedics, Walsall Manor Hospital, Walsall, GBR

**Keywords:** musculoskeletal infection, septic arthritis, bone and joint

## Abstract

Septic arthritis remains an orthopaedic emergency that requires prompt diagnosis and management. During the 2020 COVID-19 pandemic, British Orthopaedic Association (BOAST) guidelines dictated that medical treatment (closed-needle aspiration + antibiotic therapy) should be offered to patients as first-line management, and operative treatment (arthroscopic joint washout +/- synovectomy) be reserved for patients exhibiting signs of sepsis. Literature has previously shown that for native joint septic arthritis, operative treatment is not superior to medical treatment.

During the COVID-19 ‘lock-down’ period (March 2020 to June 2020), we prospectively followed the presentation, diagnosis, management and outcome of a total of six patients who presented with confirmed native joint septic arthritis.

All six patients underwent initial medical management of their septic arthritis following their diagnostic aspiration, which involved serial closed-needle aspirations and antibiotic therapy as advised by our microbiology team. Four patients went on to have an arthroscopic washout at an average of eight days following admission (mean 2.5), prior to a consultant-led decision to proceed to arthroscopic washout. The decision for operative management was the patient’s clinical deterioration based on physiological (fever, tachycardia) and biochemical (C-reactive protein (CRP), white blood cell (WBC)) parameters. All of the four patients that proceeded to operative treatment failed to provide culture yield at the time of arthroscopic washout. The mean time to discharge was 15.6 days, whilst the mean time to discharge following operative intervention was 12 days. One patient passed away during admission and one patient required a second arthroscopic washout.

Medical management of septic arthritis may play a role in symptom control in the palliative setting or in patients where a general anaesthetic is undesirable. We found operative management to be therapeutic clinically, haemodynamically and biochemically as well as facilitative of a faster recovery and shorter inpatient stay.

## Introduction

Native joint septic arthritis remains an orthopaedic emergency that requires high clinical suspicion, prompt diagnosis and management. Sharff et al. have previously shown that delayed or incomplete treatment may result in irreversible joint destruction and increased patient morbidity [1]. In the worst cases, native joint septic arthritis carries a fatality rate of 5%-15% [2]. Despite previous research indicating that medical management (closed needle aspiration plus antibiotic therapy) is not inferior to surgical management (arthroscopic +/- open joint washout and antibiotic therapy) [3-4], the favoured management of native joint septic arthritis in our centre is that of surgical management, involving an initial diagnostic joint aspirate, tailored antibiotics guided by our microbiology team and arthroscopic washout out +/- synovectomy. During the COVID-19 pandemic, the British Orthopaedic Association (BOAST 2020) released orthopaedic guidelines to match the context of the global pandemic and account for a national decrease in theatre capacity, as well as the desired avoidance of unnecessary procedures requiring a general anaesthetic and intubation. The treatment of confirmed native joint septic arthritis, therefore, included serial closed needle aspiration to dryness in combination with intravenous anti-biotics guided by a microbiology team and diagnostic aspirate growth. Operative management was reserved for those patients who exhibited signs of sepsis.

## Materials and methods

Between March 28, 2020, and June 31, 2020, we prospectively tracked six patients who presented to our major trauma centre (MTC) with proven native joint septic arthritis. Their clinical progress during admission and post-discharge outcomes were recorded using our electronic note system as well as outpatient documentation recorded at any orthopaedic clinic follow-up appointment. The collectors were made up of junior doctors who had no influence over any clinical decision-making, which was made by the ‘on the day’ trauma and orthopaedic consultant, not involved in the study. Departmental ethical approval was sought and approved with reference CARMS-16222.

## Results

Between March 28, 2020, and May 31, 2020, six patients presented to our MTC with a proven native joint septic arthritis. Four were male and two were female, with an average age of 60 (49-74). All cases involved the knee joint (four left and two right). Following diagnostic aspirate, two patients were culture positive for Escherichia coli, one patient grew methicillin-susceptible Staphylococcus aureus (MSSA) and three patients grew Group A beta-haemolytic Streptococcus. Two patients presented with disseminated infection, both of which grew Group A beta-haemolytic Streptococcus; all were haemodynamically stable on admission.

The mean number of serial closed needle aspiration to dryness (not including diagnostic admission aspirate) for all patients was 2.5 (range 1-4). Four patients subsequently went on to have an arthroscopic washout at a mean of eight days following admission (range 2-14), one patient required two arthroscopic washouts (Day 2 and Day 11 following admission). Of the two patients who did not proceed to arthroscopic washout, one died (this patient had a past medical history of palliative gastric carcinoma, and serial aspiration was used for symptom control) and one was discharged once biochemically and clinically stable.

The average inpatient hospital stay of patients who were eventually discharged was 15.6 days (7-23); the average stay in hospital following arthroscopic washout was 9.7 (3-21). Failure of serial aspiration was defined as worsening biochemical markers (CRP and WBC), worsening physiological markers (febrile) and or worsening clinical examination (pain, swelling, joint irritability, inability to weight bare). The decision to proceed to an arthroscopic washout was consultant-led, all of which were blind to the study. All patients who underwent arthroscopic washout failed to yield any bacterial culture from their intra-operative sampling. All patients were treated with intravenous (IV) antibiotics, which was tailored by our microbiology team following their diagnostic aspirate. Following their inpatient management, all patients were followed up in a face-to-face fracture clinic and none, to date, have required any further intervention. (data summarised in Table 1).

**Table 1:** Patient demographics MSSA: methicillin-susceptible Staphylococcus aureus

Patient Number	Sex	Age	COVID status	Growth	Number of aspirates	Admission to washout (Days)	Number of arthroscopic washouts	Inpatient stay (Days)
1	M	61	Negative	MSSA	3	14	1	17
2	M	74	Negative	E. Coli	3	NA	0	Died
3	M	49	Negative	Beta Haemolytic Strep	4	8	1	17
4	M	54	Negative	Beta Haemolytic Strep	1	2 & 11	2	23
5	M	73	Negative	E. Coli	2	NA	0	7
6	F	51	Negative	Beta-Haemolytic Strep	2	8	1	14

## Discussion

Research into the direct comparison between the medical management and operative management of native joint septic arthritis is scarce. However, the medical management of septic arthritis is not a novelty, with good results being seen in treating native hip septic arthritis with serial aspiration in the paediatric population, and this remains a validated treatment option [5]. Recent research has indicated that in the adult population, the medical management of native joint septic arthritis is not inferior to surgical management [3,6] but has failed to demonstrate any patient outcome or cost advantages. Literature also fails to define a medical management timeline, particularly in regards to the timing of serial aspiration, the number of aspirates to be therapeutic, and objective measurements to assess the effectiveness of treatment.

The favoured practice at our MTC consists of initial diagnostic joint aspiration, tailored antibiotics guided by our microbiology team (with specified mode and duration of delivery), and arthroscopic washout +/- synovectomy. During the COVID pandemic and the introduction of the COVID BOAST guidelines, our centre used medical management as a first-line option in treating patients with native joint septic arthritis, in the absence of sepsis.

Of our small series of patients who presented with septic arthritis and received first-line medical management, the majority went on to have an arthroscopic washout of their joint following a consultant-led decision of physiological/biochemical or clinical patient deterioration. Whilst this may seem like a failure of medical management, there may be an element of ‘surgical impatience’, with previous literature suggesting that the time to sterilisation of synovial fluid, with the use of serial aspiration, can take between four and eight days following the onset of antibiotic therapy [7], with the average time to arthroscopic washout being eight days (range 2-14) in our series. Furthermore, there is no clear definition of ‘serial’ in regards to aspiration technique, with some studies using daily aspiration whilst others use fluid re-accumulation as an indication for the next aspirate to dryness [7]. Our series received serial aspiration with timing being limited in some cases by patient compliance and consent (with patients reporting this procedure as being painful and unpleasant). Interestingly, of the patients in our series who progressed to arthroscopic washout, all failed to yield any bacterial culture from their intra-operative sampling. It is difficult to conclude whether this was due to serial aspiration having a therapeutic effect or, more likely, the presence of antibiotic therapy. Although the efficiency of treatment for septic arthritis may be debated, the length of treatment and thus inpatient hospital stay is not, with all previous research indicating a longer length of stay in medically managed septic arthritis [3,6,8], to which our series is no exception. In our case series, we used serial aspiration for one patient as a mode of palliative symptom control. The decision for palliation was based on their complex and unrelated past medical history and serial aspiration was observed, from the clinical noting, to provide the patient with symptomatic relief.

Limitations

A large limitation of this case series is population size. Given serial aspiration is not the first-line management in our MTC, the collection period was limited to the COVID-19 pandemic and the surgical restrictions that came with it. Our definition of ‘serial aspiration’ for this study was every 24 hours; however, this did not happen in some instances, largely due to patient compliance and consent, which may have affected the outcome. Although the lead consultants making the decision of arthroscopic washout were unaware of the study, this was not a randomised control trial and, therefore, lacks power. A review of patients also took place by a different consultant each day, whose threshold for arthroscopic washout may have differed, introducing potential bias.

## Conclusions

Whilst the medical management of septic arthritis may play a therapeutic role in the context of the COVID-19 pandemic with the desire to avoid a general anaesthetic or in the palliative setting for symptom control, we found operative management to be therapeutic clinically, haemodynamically and biochemically, as well as facilitative of a faster recovery and shorter inpatient stay. More research, particularly randomised control trials, out of the pandemic, need to be conducted to ascertain if the medical management of native joint septic arthritis is equivalent to surgical management with regards to patient outcomes, cost and length of stay.
